# Identification of Novel Variants of Metadherin in Breast Cancer

**DOI:** 10.1371/journal.pone.0017582

**Published:** 2011-03-08

**Authors:** Xianqiang Liu, Ning Zhang, Xiao Li, Meena S. Moran, Cunzhong Yuan, Shi Yan, Liyu Jiang, Tingting Ma, Bruce G. Haffty, Qifeng Yang

**Affiliations:** 1 Department of Breast Surgery, Qilu Hospital, School of Medicine, Shandong University, Ji'nan, Shandong, People's Republic of China; 2 Department of Surgery, Affiliated Jinan Central Hospital of Shandong University, Ji'nan, Shandong, People's Republic of China; 3 Obstetrics and Gynecology, Qilu Hospital, School of Medicine, Shandong University, Ji'nan, Shandong, People's Republic of China; 4 Department of Therapeutic Radiology, Yale University School of Medicine, New Haven, Connecticut, United States of America; 5 Department of Radiation Oncology, UMDNJ-Robert Wood Johnson School of Medicine, and the Cancer Institute of New Jersey, New Brunswick, New Jersey, United States of America; University of California, Los Angeles, and Cedars-Sinai Medical Center, United States of America

## Abstract

Metadherin (MTDH, also known as AEG-1, and Lyric) has been demonstrated to play a potential role in several significant aspects of tumor progression. It has been reported that overexpression of MTDH is associated with progression of disease and poorer prognosis in breast cancer. However, there are no studies to date assessing variants of the MTDH gene and their potential relationship with breast cancer susceptibility. Thus, we investigated all variants of the MTDH gene and explored the association of the variants with breast cancer development. Our cohort consisted of full-length gene sequencing of 108 breast cancer cases and 100 healthy controls; variants were detected in 11 breast cancer cases and 13 controls. Among the variants detected, 9 novel variants were discovered and 2 were found to be associated with the susceptibility of breast cancer. However, additional studies need to be conducted in larger sample sizes to validate these findings and to further investigate whether these variants are prognostic in breast cancer patients.

## Introduction

Metadherin (MTDH, also known as AEG-1, and Lyric), was first reported in 2002 as a novel late response gene following HIV-1 infection or treatment with recombinant HIV-1 envelope glycoprotein (gp120) [1]. The full-length cDNA has subsequently been independently cloned by four different groups of investigators [2], [3], [4], [5]. A “lung homing domain”, which mediates lung metastasis in the 4T1 mouse mammary tumor cell, was identified in MTDH in 2004 with phage display screening and was subsequently named metadherin [4]. In addition, the mouse-rat MTDH was also found to serve as a junction protein [5] and was found to be located in the cytoplasm, endoplasmic reticulum, perinuclear regions, and nucleolus [3].

Kang and colleagues first cloned and characterized the human full-length MTDH gene; it was found to encode a single-pass transmembrane protein with a calculated molecular mass of 64 KDa, containing 12 exons and 11 introns with a full-length of 86,082 bp, and a cDNA of 3611 bp (excluding the poly-A tail) [2]. Subsequently, the human MTDH gene was localized at chromosome 8q22 and has been reported to be amplified in a number of malignancies such as malignant glioma [6], hepatocellular carcinoma (HCC)[7], and breast cancer [8], [9], [10]. Our group has also previously published on MTDH, using computational algorithms to establish the overexpression of MTDH in breast cancer [11].

Following its initial identification, MTDH was thought to be a potential focus for targeted therapy based on its multi-facet roles in regulating cancer progression. Though the MTDH gene was cloned only 5 years ago, this novel gene is now known to be a potent mediator in the development of malignancies and component of oncogenic signaling pathways. MTDH has been demonstrated to play a role in several significant stages of tumor progression, including transformation, initiation of apoptosis, invasion, metastasis, chemoresistance and angiogenesis. Furthermore, recent studies have established its performance in PI3K/Akt, nuclear factor κB (NFκB), and Wnt/β-catenin signaling pathways, laying its foundation as a feasible potential target, both clinically and experimentally, for future targeted therapy development.

Since breast cancer has become the most common tumor among women in the whole world [11], and MTDH has been demonstrated to be critically involved in breast cancer progression and metastasis by our group and others,[4], [12], [13], [14], [15], in this study, we attempted to further investigate the potential role of MTDH in breast cancer development.

Single nucleotide polymorphisms (SNP) are defined as genetic variation in a DNA sequence of a particular gene when a single nucleotide is altered; SNPs are usually considered to be point mutations that have been evolutionarily successful enough to recur in a significant proportion of the population and can occur in coded or non-coded regions, thus the variant may or may or may not affect the function of the gene. Identification of SNPs in the host can potentially facilitate the evaluation of the susceptibility of cancer or predict progression of disease of response to treatment. Here, we investigated the genetic polymorphisms in MTDH by direct sequencing in a cohort of breast cancer cases and controls, with the intention of discovering of novel variants and comparing the distribution of SNP genotype frequencies of these 2 affected vs. non-affected cohorts to determine whether a particular SNP may influence susceptibility to breast cancer development.

## Materials and Methods

### Patients and Sample

The study involved 108 breast cancer patients diagnosed with breast cancer in Qilu Hospital (Shandong, China) between September 2008 and April 2010. All breast cancer cases were classified according to the WHO classification of breast tumors. The breast cancer cohort was histologically comprised of 7 cases of lobular carcinoma, 82 cases of ductal carcinoma and 19 classified as other malignant sub-types of breast cancer. For the control group, 100 healthy women unaffected with breast cancer were included to investigate SNPs as susceptibility biomarkers. Most of the patients and controls were Han nationality resides in Shandong Provence, China. Details of the patient characteristics for the 2 cohorts are shown in Table 1. For all participants in this study, written informed consent was obtained as delineated by the protocol which was approved by the Ethical Committee of Shandong University. All patients donated 1.5 ml of whole blood which was stored at −80°C in our university laboratory.

**Table 1:** Clinical data of cases and controls.

Clinical data	Cases	Controls
Age (Mean±SD) a	48.9±10.4	45.2±8.9
rade (%)		
I,II	59(79.7)	_
III	15(20.3)	
ER (%)		
Negative	28(26.9)	_
Positive	76(73.1)	
PR (%)		
Negative	37(35.9)	_
Positive	66(64.1)	

a The Student's t test showed no significant difference between the two groups (p>0.05).

### DNA extraction

DNA from each sample of whole blood was extracted with the TIANamp Genomic DNA Kit (Tiangen, Beijing, China) as directed in the manufacturer's instructions. The concentration of DNA and the purity of each sample were measured by ultraviolet spectrophotometer (GE Healthcare, USA). DNA samples were routinely stored at −20°C.

### Sequencing analysis of human MTDH gene

The full-length MTDH gene was sequenced to investigate genetic variants in DNA samples from the 108 cases and 100 controls. The MTDH gene was amplified with polymerase chain reaction (PCR) prior to sequencing. In each 50 µl PCR reaction, 1 µl genomic DNA (100 ng/µl) was amplified using 1 U EasyTaq DNA polymerase (Transgen, Beijing, China) with 4 µl of 2.5 mM dNTPs and 2 µl of each primer. The PCR conditions were as follows: 94°C for 5 min, 35 cycles of 94°C for 30 seconds, individual annealing temperature for 30 seconds, 72°C for 30 seconds and a final extension step of 72°C for 10 min. The 13 primer sets of MTDH used for the amplification and sequencing analysis are shown in Table 2 (GenBank sequence; NC_000008.10 released in JUNE 10, 2004). All sequencing data was produced by the Genomic Analysis Facility (BioSune, Shanhai, China) and analyzed with Meglign 7.0 and Chromas 2.33 software.

**Table 2:** Thirteen primer sets of MTDH for PCR amplification.

No.	Primer	Region	Sequence of primer	Length
1	MTDHUTR1-F	5′ UTR	5′CTGGCAACTGGTAGGCACGC3′	893 bp
2	MTDHUTR1-R		5′GAGGGACTCGCAGGATGACG3′	
3	MTDH1-F	Exon 1	5′ACCTTCCCTGACACGCCTTTG3′	852 bp
4	MTDH1-R		5′CTCTACTGCCGCTCATCCCATAC3′	
5	MTDH2-F	Exon 2	5′TGTAGAAATAAGGCTCGGAGAC3′	1031 bp
6	MTDH2-R		5′GGCTCAAGCAATCTACCCAC3′	
7	MTDH34-F	Exon 3&4	5′CTTTGGAGCCAGACAGACCTA3′	1553 bp
8	MTDH34-R		5′GACTCAAGGCAGAAAGGCAAAT3′	
9	MTDH5-F	Exon 5	5′CCTAAGTCCTGGTCCCAAAT3′	1101 bp
10	MTDH5-R		5′GCCTGAAAGAAGAAGGTGCT3′	
11	MTDH6-F	Exon 6	5′GGCTCCACCACAAAGATTCA3′	856 bp
12	MTDH6-R		5′TTAATTCCCACCTTGCTCTAC3′	
13	MTDH7-F	Exon 7	5′AAAGTCTCCGTGTCAAAGTATAG3′	647 bp
14	MTDH7-R		5′TTTCCGAACATACAAACCAT3′	
15	MTDH8-F	Exon 8	5′GAGTGGAATCAAATGGCTAAT3′	934 bp
16	MTDH8-R		5′CATAGTGTCGGGCTAGTAATC3′	
17	MTDH9-F	Exon 9	5′TGTTAGCCAGGATGGTCTCG3′	936 bp
18	MTDH9-R		5′AAATGTCTGTTGGGTAGATGC3′	
19	MTDH10-F	Exon 10	5′GGCAATTCTCATACCTCCTC3′	1154 bp
20	MTDH10-R		5′ATGTCTAAGCTGTCTATCCCTT3′	
21	MTDH11-F	Exon 11	5′TGATGATTGATTTGGCTGTA3′	738 bp
22	MTDH11-R		5′AAAGGAAGAAAGGGCTACT3′	
23	MTDH12-1-F	Exon 12	5′AAGGAGGGAAGAAGACATAG3′	1249 bp
24	MTDH12-1-R		5′TCTACGCACTACAGGTTAAG3′	
25	MTDH12-2-F	Exon 12	5′AGATTGTGCCCTATCTCATCT3′	1415 bp
26	MTDH12-2-R		5′AGAAATTCATCCTTGGCTCT3′	

### Statistical Analysis

The genotypes and allele frequencies of the known and novel SNPs were tested for equilibrium using a validated, public statistical web-tool based on the Hardy-Weinberg model [16] with a p value of >0.5 cm suggesting equilibrium. The distribution of known and novel SNPs between the case and control groups were analyzed using chi-squared and the Fisher's exact test was used when one cell count was less than 5. All p values were exacted two-sided and p values of <0.05 were considered statistically significant. Odds ratios (OR) and 95% confidence intervals (CIs) were calculated using unconditional logistic regression analysis to evaluate the association between genotype frequencies and risk of breast cancer development. The statistical analyses were conducted using SPSS Software 17.0 (SPSS Inc. Chicago, Illinois, USA).

## Results

### Novel variants discovered in MTDH and the characterization

The patients in both cohorts (affect and control) were all from mainland China; there were no significant clinical differences (i.e. median ages, menstrual history, body mass index [BMI] or other related parameters) for the 2 cohorts.

**Figure 1:**
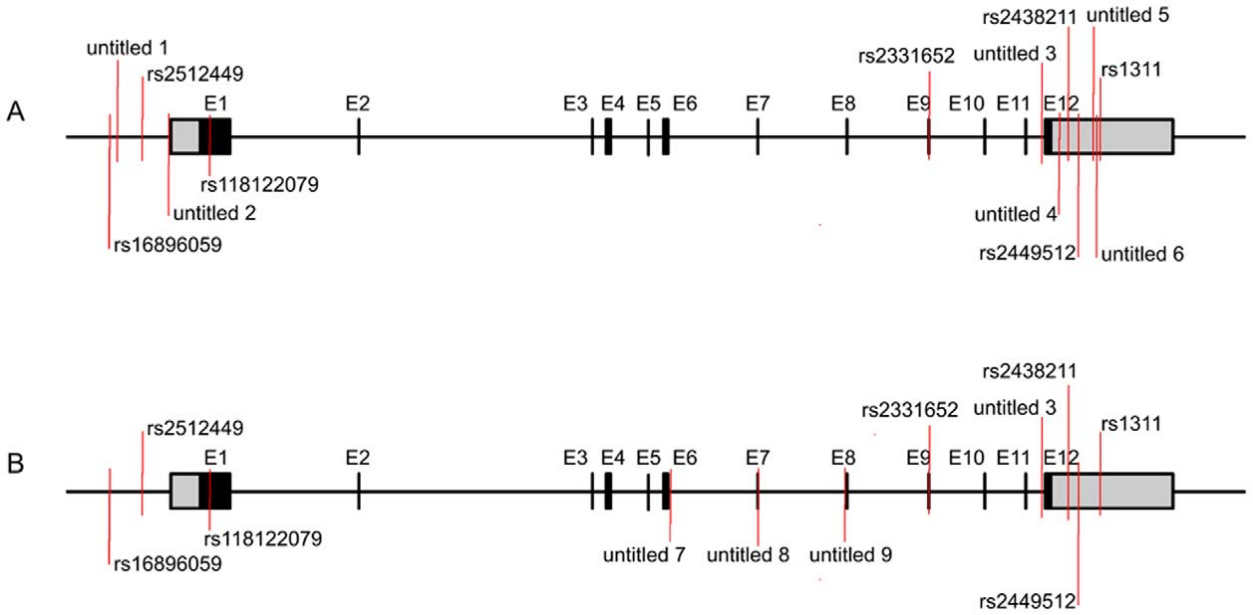
The distribution of detected variants of MTDH. The black blocks stand for the 12 exons and the grey blocks represent for the 3′UTR and 5′UTR respectively. The red line marked the location of each variant in MTDH. A. Control group. B. Case group.

Thirteen variants were detected in the control group compared with eleven variants in the affected breast cancer cohort; of these, 9 unnamed, novel variants were discovered in sum. The distribution of all variants in both control and case groups is displayed in Figure 1. The novel and known variants appear to gather at both ends of MTDH gene, with 3 novel variants displayed in the central part of the gene in case group, but not in the control group. The 3 variants are located at exon_6 (untitled_7), exon_7 (untitled_8) and intron_7 (untitled_9) individually.

**Figure 2:**
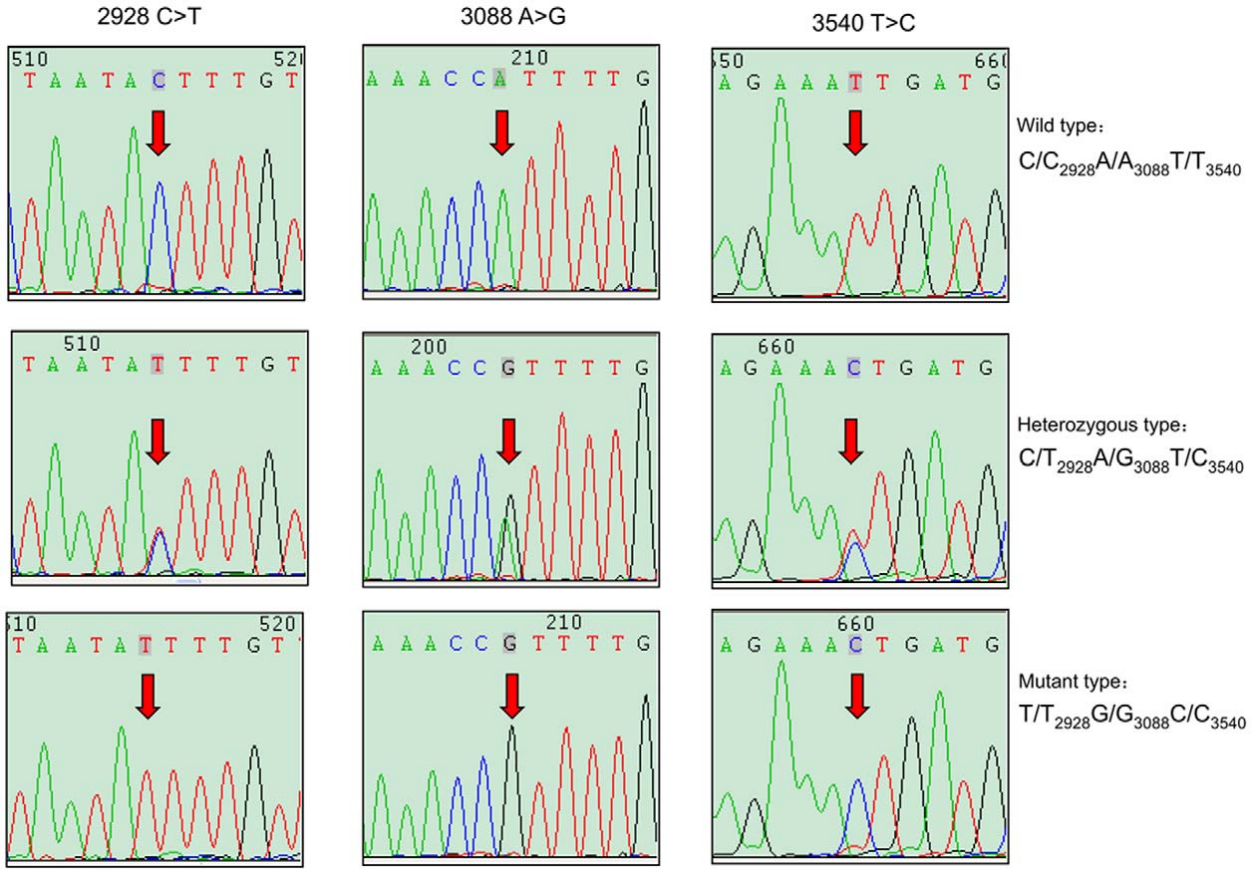
Sequencing chromatogram results of the complete linkage among rs2438211, rs2449512 and rs1311.

Among all the variants detected, 3 SNPs (rs2438211, rs2449512 and rs1311) were found with complete linkage in both groups; the sequencing chromatogram results representing the linkage are shown in figure 2. In addition to the known SNPs, several new SNPs were discovered: one novel variant in the flanking sequence; one in the 5′UTR; three in the 3′UTR; and one in intron 11 for the control group. Of note, the high frequency variant in the intron 11 was also detected in the case group. Strikingly, two low frequency novel variants were detected in case group located in exon_6 and exon_7, respectively. The variant in exon_7 (1390 G>A, untitled_8) was a synonymous SNP while the variant in exon_6 (1333 C>G, untitled_7) converted a Asp to Glu in the MTDH protein. Only one patient with breast cancer carried the SNP (G>C, untitled_9) just one nucleotide prior to exon_8, which was against the “GT-AG rule” in mRNA splicing. A detailed description and characterization of all variants identified in this study are shown in table 3.

**Table 3:** Molecular biological information of each variant among controls (n = 100) and cases (n = 108).

Location (Relative to)								
Chromsom start	mRNA start	dsSNP rs#	Region	Protein residue	Amino Acid pos.	dbSNP allele	Controls	SNP pos. c
98655937	−470	rs16896059	Flanking seq			G/A	G/G 55; A/G 34; A/A11	GGC**G**TCAG
98656002	−405	untitled_1				C/T	C/C 99; C/T 1; T/T 0	ATTC**C**ACCT
98656207	−200	rs2512449				A/G	G/G 38; A/G 43; A/A 19	GTCC**A**CCAA
98656407	1	untitled_2	5′UTR			G/A	G/G 98; A/G 2; A/A 0	TCGC**G**GCCC
98656788	382	rs118122079	exon_1	Ser-Ser	18	G/A	G/G 99; G/A 1; A/A 0	GCTC**G**GCCC a
98725970	1681	rs2331652	exon_9	Lys-Lys	451	G/A	G/G 64;A/G 31; A/A 5	AGAA**G**CAAG a
98736822	—	untitled_3	intron_11			T/C	T/T 89; C/T 9; C/C 2	TCTT**T**TTTA
98737749	2928	rs2438211	3′UTR			C/T	C/C_2928_A/A_3088_T/T_3540_53;	AATA**C**TTTG b
98737909	3088	rs2449512				A/G	C/T_2928_A/G_3088_T/C_3540_40;	ATCA**A**TTTC b
98738361	3540	rs1311				T/C	T/T_2928_G/G_3088_C/C_3540_7	AAAA**T**GGTT b
98737499	2678	untitled_4				G/T	G/G 99; G/T 1; T/T 0	AAAT**G**CAGTT
98738288	3467	untitled_5				C/T	C/C 99; C/T 1; T/T 0	CCATC**C**AATG
98738324	3503	untitled_6				A/T	A/A 99; A/T 1; T/T 0	ACAGT**A**TTGC
							**Cases**	
98655937	−470	rs16896059	Flanking seq			G/A	G/G 60; A/G 41; A/A 7	CGGC**G**TCAG
98656207	−200	rs2512449				A/G	G/G 46; A/G 47; A/A 15	GTCC**A**CCAA
98656788	382	rs118122079	exon_1			G/A	G/G 105; A/G 2; A/A 1	GCTC**G**GCCC
98703373	1333	untitled_ 7	exon_6	Asp-Glu	335	C/G	C/C 107; C/G 1; G/G 0	AAAAGA**C**TGG a
98711995	1390	untitled_ 8	exon_7	Glu-Glu	354	G/A	G/G 107; G/A 1;A/A 0	CTGA**G**CCAGT a
98718853	—	untitled_ 9	intron_7			G/C	G/G 107; G/C 1; C/C 0	TTTA**G**ATGGT
98725970	1681	rs2331652	exon_9	Lys-Lys	451	G/A	G/G 52;A/G 46;A/A 10	AGAA**G**CAAG a
98736822	—	untitled_ 3	intron_11			T/C	T/T 84; C/T 24; C/C 0	TCTT**T**TTTA
98737749	2928	rs2438211	3′UTR			C/T	C/C_2928_A/A_3088_T/T_3540_59;	AATA**C**TTTG b
98737909	3088	rs2449512				A/G	C/T_2928_A/G_3088_T/C_3540_38;	ATCA**A**TTTC b
98738361	3540	rs1311				T/C	T/T_2928_G/G_3088_C/C_3540_11;	AAAA**T**GGTT b

a The double-underlined characters standed for the codon which contains SNP.

b The complete linkage among the three SNPs (rs2438211, rs2449512 and rs1311).

c The SNP position was emphasized in bolding characters.

### The relationship between SNP genotype distribution and breast cancer susceptibility

The genotype distributions of the high frequency polymorphisms in the case and control groups are shown in table S1. When analyzed separately, the untitled_3 was found to be associated with breast cancer development significantly more frequently in the control group (p = 0.008). Furthermore, significant differences was also observed in comparing the T/T genotype to the C/C + C/T genotypes among cases and controls (p = 0.041; OR = 2.286; 95% CI: 1.054–4.995), indicating that the homozygous T/T genotype increases the risk of breast cancer development 2.286 times among Chinese women.

Additionally, the MTDH (1681 G>A, rs2331652) polymorphism was also found to increase susceptibility for breast cancer development. Amongst the case group, the A/A genotype was detected with a higher frequency than that in the control group; however, this difference was not statistically significant when analyzed as a separate genotype (p = 0.062). Nevertheless, when combined with the A/G genotype, the differences between the case and control groups were significant. (p = 0.026; OR = 1.915; 95% CI: 1.098–3.339). No significant differences in the analysis were noted when the data were re-analyzed with stratification by clinical stage and receptor status (ER and PR), suggesting the two polymorphisms may be applied in the generalized breast cancer population.

However, no significant correlations were noted amongst these variants (listed in table S1) and the clinical-pathologic parameters (age at diagnosis, pathological diagnosis, tumor size and grade, nodal metastasis, etc.), suggesting that these variants may not be prognostic indicators in female breast cancer (Data not shown). The haplotype frequency of rs2331652 and untitled_3 was listed in table S2; however, no significant linkage was found between the two variants which met statistical significance associated with breast cancer.

## Discussion

Recently, a number of studies have elucidated the multi-faceted role of MTDH in tumor progression. Overexpression of MTDH has been observed in carcinomas and has been correlated with poor clinical outcomes [12], [15]. As a gene located downstream to Ha-Ras and c-Myc, MTDH mediates the transforming activity of both oncogenes [17] and furthermore, is involved multiple signaling pathways including the PI3K/Akt [17], [18], nuclear factor κB(NFκB) [19], and Wnt/β-catenin pathways [20]. Thus, MTDH regulates different aspects of cell activity and may function as a biomarker for predicting disease progression and responses to different treatments [21]. Given the recent interest in the MTDH gene and its suggested important role in the development of breast cancer, this study was conducted to investigate both known and novel SNPs to analyze if any of these variants of MTDH contribute to the risk of breast cancer development.

In our present study, 13 variants in control group and 11 in case group were detected. Several of these variants are being described for the first time in this publication. Among these novel variants, MTDH (1333 C>G, untitled_7) polymorphism cause an Asp to Glu conversion in MTDH protein, which may facilitate the structural research of the protein. Interestingly, one patient with breast cancer carried a G/C variant (C>G, untitled_9) just one nucleotide prior to exon_8, which was against the “GT-AG rule” in mRNA splicing Therefore, this single nucleotide conversion will cause the failure in splicing of mtdh protein and subsequently dysfunction of this protein.

In this study, two variants were identified that were significantly associated with increased susceptibility for breast cancer development. Significant difference was found between case and control group in both untitled_3 and MTDH (1681 G>A, rs2331652) polymorphism; these variants require functional analysis and validation in larger patient populations to confirm the correlation to breast cancer susceptibility. Furthermore, the association of these variants to outcomes needs to be examined to determine if these SNPs are also prognostic indicators for disease recurrence or progression in breast cancer patients.

As indicated previously, the participants involved in our study were mainly residents in Shandong Province, China. Since the Chinese population generally is genetically more homogeneous than other ethnic populations, we predict similar findings in larger sample sizes across China, but the applicability of our findings to other ethnic populations (both within Asian and outside Asia) will need further investigation in varying patient populations before these data can be generally extrapolated to other ethnicities.

The discrepancy between the lack of correlation of the variants in the current study to disease progression in breast cancer (data not shown), and the previously documented [12] high MTDH expression contributing to increased metastasis and poor prognosis warrants further discussion. The two studies were comprised of completely different ethnic groups (one study had U.S. citizens with significant genetic heterogeneity, while the current study was more homogeneous with all participants of Chinese descent); thus, ethnicity/race may be an independent factor affecting the association of the distribution of MTDH SNPs and breast cancer development. This highlights the need for multi-ethnic evaluation of MTDH in future studies.

In conclusion, in addition to finding some known variants of MTDH, several novel SNPs of MTDH were discovered in our study that appear to be correlated to breast cancer susceptibility Since MTDH has been demonstrated as a key regulator in the complex network of oncogenic pathways, additional investigations further clarifying the functional role of this gene and its association to breast cancer development and recurrence are needed. While additional study on the MTDH gene is necessary, variants of MTDH may prove to be potential markers for breast cancer development, prognostic indicators for disease progression, and possible foci for future targeted therapy.

## Supporting Information

Table S1 The genotype distribution of SNPs in MTDH in cases and controls.
(DOC)

Table S2 Hyplotype frequency a of rs2331652 and untitled_ 3.
(DOC)
