# Supplementary material for: Identification of Novel Variants of Metadherin in Breast Cancer
Source: PLoS One. 2011 Mar 8;6(3):e17582. doi: 10.1371/journal.pone.0017582 (PMC3050918; doi:10.1371/journal.pone.0017582)
Supplement: Table S1 — The genotype distribution of SNPs in MTDH in cases and controls. (DOC) [file pone.0017582.s001.doc]

| **Table S1. The genotype distribution of SNPs in MTDH in cases and controls** | | | | | |
| --- | --- | --- | --- | --- | --- |
| **dsSNP rs#** | **Genotypes** | **No. of subjects** | | ***p* value^a^** | **OR (95%CI)^b^** |
|  |  | **Controls** | **Cases** |  |  |
| rs16896059 | G/G | 55 | 60 | 0.502 | 1 |
|  | A/G | 34 | 41 |  | 1.105(0.617-1.981) |
|  | A/A | 11 | 7 |  | 0.583(0.211-1.611) |
|  |  |  |  |  |  |
|  | G/G | 55 | 60 | 1.000 | 1 |
|  | A/A+A/G | 45 | 48 |  | 0.978(0.566-1.690) |
|  |  |  |  |  |  |
|  | A/A | 11 | 7 | 0.325 | 1 |
|  | G/G+A/G | 89 | 101 |  | 0.561(0.208-1.508) |
|  |  |  |  |  |  |
| rs2512449 | G/G | 38 | 46 | 0.574 | 1 |
|  | A/G | 43 | 47 |  | 0.903(0.497-1.639) |
|  | A/A | 19 | 15 |  | 0.652(0.293-1.454) |
|  |  |  |  |  |  |
|  | G/G | 38 | 46 | 0.572 | 1 |
|  | A/A+A/G | 62 | 62 |  | 0.826(0.474-1.440) |
|  |  |  |  |  |  |
|  | A/A | 19 | 15 | 0.352 | 1 |
|  | G/G+A/G | 81 | 93 |  | 0.688(0.328-1.441) |
|  |  |  |  |  |  |
| rs118122079 | G/G | 99 | 105 | 1.000 | 1 |
|  | A/G | 1 | 2 |  | 1.886(0.168-21.124) |
|  | A/A | 0 | 1 |  | N/A |
|  |  |  |  |  |  |
|  | G/G | 99 | 105 | 0.622 | 1 |
|  | A/A+A/G | 1 | 3 |  | 2.829(0.289-27.648) |
|  |  |  |  |  |  |
|  | A/A | 0 | 1 | 1.000 | 1 |
|  | G/G+A/G | 100 | 107 |  | N/A |
|  |  |  |  |  |  |
| rs2331652 | G/G | 64 | 52 | 0.062 | 1 |
|  | A/G | 31 | 46 |  | 1.826(1.018-3.276) |
|  | A/A | 5 | 10 |  | 2.462(0.792-7.652) |
|  |  |  |  |  |  |
|  | G/G | 64 | 52 | 0.026* | 1 |
|  | A/A+A/G | 36 | 56 |  | 1.915(1.098-3.339) |
|  |  |  |  |  |  |
|  | A/A | 5 | 10 | 0.289 | 1 |
|  | G/G+A/G | 95 | 98 |  | 1.939(0.639-5.883) |
|  |  |  |  |  |  |
| untitled_3 | T/T | 89 | 84 | 0.008* | 1 |
|  | C/T | 9 | 24 |  | 2.794(1.228-6.358) |
|  | C/C | 2 | 0 |  | N/A |
|  |  |  |  |  |  |
|  | T/T | 88 | 94 | 0.041* | 1 |
|  | C/C+C/T | 11 | 24 |  | 2.286(1.054-4.995) |
|  |  |  |  |  |  |
|  | C/C | 2 | 0 | 0.228 | 1 |
|  | T/T+C/T | 97 | 108 |  | N/A |
|  |  |  |  |  |  |
| rs2438211^c^ | T/T | 7 | 11 | 0.632 | 1 |
|  | C/T | 40 | 38 |  | 0.605(0.212-1.722) |
|  | C/C | 53 | 59 |  | 0.708(0.256-1.960) |
|  |  |  |  |  |  |
|  | T/T | 7 | 11 | 0.467 | 1 |
|  | C/C+C/T | 93 | 97 |  | 0.664(0.247-1.785) |
|  |  |  |  |  |  |
|  | C/C | 53 | 59 | 0.889 | 1 |
|  | T/T+C/T | 47 | 49 |  | 1.068(0.619-1.843) |

^a^ Two-sided χ^2^ test for distribution of separate genotypes and combined genotypes between case and control group.

^b^ OR(95%CI) was obtained from unconditional logistic regression analysis.

^c^ rs2438211, rs2449512 and rs1311 were found in complete linkage in MTDH gene, and only rs2438211 were displayed to represent for the genotype frequency of this linkage.
