# Supplementary material for: Identification of Novel Variants of Metadherin in Breast Cancer
Source: PLoS One. 2011 Mar 8;6(3):e17582. doi: 10.1371/journal.pone.0017582 (PMC3050918; doi:10.1371/journal.pone.0017582)
Supplement: Table S2 — Hyplotype frequency a of rs2331652 and untitled_ 3. (DOC) [file pone.0017582.s002.doc]

| **Table S2. Hyplotype frequency ^a^ of rs2331652 and untitled_ 3** | | | |
| --- | --- | --- | --- |
| **Hyplotype** | | **Controls** | **Cases** |
| **rs2331652** | **untitled_3** |
| G/G | T/T | 55 | 37 |
| G/G | T/C | 7 | 15 |
| G/G | C/C | 2 | 0 |
| A/G | T/T | 29 | 37 |
| A/G | T/C | 2 | 9 |
| A/A | T/T | 5 | 9 |
| A/A | T/C | 0 | 1 |
| Total | | 100 | 108 |

^a^ No significant linkage was found between the two variants.
